# The Scientific American

**Published:** 1878-12

**Authors:** 


*The Scientific American.*—Thirty-third Year.

This widely circulated and splendidly illustrated paper is published weekly. Every number contains sixteen pages of useful information, and a large number of original engravings of new inventions and discoveries, representing Engineering Works, Steam Machinery, New Inventions, Novelties in Mechanics, Manufactures, Chemistry, Electricity, Telegraphy, Photograpy, Architecture, Agriculture, Horticulture, Natural History, etc.

All Classes of Readers find in the *Scientific American* a popular *resume* of the best scientific information of the day; and it is the aim of the publishers to present it in an attractive form, avoiding as much as possible abstruse terms. To every intelligent mind, this journal affords a constant supply of instructive reading. It is promotive of knowledge and progress in every community where it circulates.

*Terms of Subscription.*—One copy of the *Scientific American* will be sent for *one year*—52 numbers—postage prepaid, to any subscriber in the United States or Canada, on receipt of three dollars and twenty cents by the publishers; six months, $1.60; three months, $1.00.

*Clubs.*—One extra copy of the *Scientific American* will be supplied gratis *for every club of five subscribers* at $3.20 each; additional copies at same proportionate rate. Postage prepaid.

One copy of the *Scientific American* and one copy of the *Scientific American Supplement* will be sent for one year, postage prepaid, to any subscriber in the United States or Canada, on receipt of *seven dollars* by the publishers.

The safest way to remit is by postal Order, Draft, or Express. Money carefully placed inside of envelopes, securely sealed, and correctly addressed, seldom goes astray, but is at the sender's risk. Address all letters and make all orders, drafts, etc., payable to Munn & Co., 37 Park Row, New York.

*To Foreign Subscribers.*—Under the facilities of the Postal Union, the *Scientific American* is now sent by post direct from New York, with regularity, to subscribers in Great Britian, India, Australia, and all other British colonies; to France,

Austria, Belgium, Germany, Russia, and all other European States; Japan, Brazil, Mexico, and all States of Central and South American. Terms, when sent to foreign countries, Canada excepted, $4; gold, for *Scientific American*, 1 year; $9, gold, for both *Scientific American* and *Supplement* for 1 year. This includes postage, which is prepaid. Remit by postal order or draft to order of Munn & Co., 37 Park Row, New York.

